# PTD-FNK Alleviated Heat Stress-Induced Apoptosis of Boar Sertoli Cells via the PI3K/AKT Pathway

**DOI:** 10.3390/vetsci13080798

**Published:** 2026-08-12

**Authors:** Qiuyan Huang, Qiqi Ma, Shiyu Yang, Yanling Wang, Bin Zheng, Weixia Ji, Xingxing Song, Xin Zhang, Xun Li, Xiaoye Wang, Sutian Wang, Chuanhuo Hu

**Affiliations:** 1 Guangxi Key Laboratory of Animal Breeding, Disease Control and Prevention, College of Animal Science and Technology, Guangxi University, Nanning 530004, China; 2 State Key Laboratory of Swine and Poultry Breeding Industry, Guangdong Provincial Key Laboratory of Animal Breeding and Nutrition, Institute of Animal Science, Guangdong Academy of Agricultural Sciences, Guangzhou 510640, China; 3 Animal Husbandry Research Institute, Guangxi Vocational University of Agriculture, Nanning 530001, China; wangyanling0705@163.com; 4 Nanning Dabei Agricultural Feed Technology Co., Ltd., Nanning 530105, China

**Keywords:** PTD-FNK, Sertoli cells, heat stress, apoptosis, PI3K/AKT pathway

## Abstract

Heat stress (HS) impairs boar reproductive capacity via damaging Sertoli cells (SCs) and disrupting the integrity of the blood–testis barrier (BTB). This present study investigated the protective effects and underlying molecular mechanisms of PTD-FNK against HS-induced injury in porcine SCs. A HS model (43 °C, 1 h) was established, and 0.1 nM was determined as the optimal working concentration of PTD-FNK. Results showed that PTD-FNK effectively reversed HS-induced BTB disruption by restoring the expression levels of tight junction proteins (Claudin-1, Occludin, ZO-1, and Cx43) to baseline levels (*p* < 0.05). Concurrently, PTD-FNK alleviated HS-induced oxidative stress by enhancing total antioxidant capacity (T-AOC) and superoxide dismutase (SOD) activity, while reducing malondialdehyde (MDA) content (*p* < 0.05). It also suppressed HS-triggered apoptosis by downregulating the expression of pro-apoptotic proteins (Caspase-3/8/9 and Bax) and upregulating the anti-apoptotic protein Bcl-2 (*p* < 0.05). Furthermore, PTD-FNK maintained cellular homeostasis by regulating mitochondrial dynamics—restoring mitochondrial membrane potential (MMP) and balancing the expression of fusion-related protein Mfn1 and fission-related protein Drp1.Transcriptomic and LY294002 experiments confirmed PTD-FNK exerted effects via the PI3K/AKT pathway, modulating ID3, H2AX, DDIT3, CDKN1C, RAD51, and TP53. Thus, PTD-FNK protects SCs from HS through multi-target regulation dependent on PI3K/AKT, providing a novel strategy for boar reproductive health under HS.

## Simple Summary

This study explored the protective mechanism of the fusion protein PTD-FNK (Protein Transduction Domain-FNK) against heat stress (HS) injury in boar Sertoli cells (SCs). We established a 43 °C 1 h HS cell model and screened 0.1 nM as the optimal PTD-FNK concentration. Results showed PTD-FNK restored blood–testis barrier integrity, alleviated oxidative stress, balanced mitochondrial dynamics, and suppressed cell apoptosis. Transcriptome sequencing combined with PI3K inhibitor LY294002 verification confirmed PTD-FNK exerted multi-target protective effects by activating the PI3K/AKT pathway. This work provides a new intervention strategy to mitigate heat-induced boar reproductive damage.

## 1. Introduction

Heat stress (HS) is a nonspecific physiological response of animals to high-temperature environments, with its core mechanism being the reduction of metabolic heat production by decreasing feed intake and redistributing blood flow [1,2]. However, this adaptive response often triggers a cascade of problems such as energy imbalance, nutritional deficiency, and reproductive dysfunction [3]. Pigs, lacking sweat glands and commonly reared in high-density environments under intensive farming systems, are particularly susceptible to HS. HS not only disrupts the water, protein, and energy metabolism of pigs and impairs hormonal balance [4,5], but also directly damages the male reproductive system, resulting in decreased semen quality, reduced libido, and even adverse effects on embryonic development, leading to decreased placental and fetal weights [6,7].

Specifically, studies have shown that when boars are exposed to ambient temperatures above 30 °C for 3 consecutive days, their sperm motility decreases by 30–40%, and the abnormal sperm rate increases by 25–35% [7]. Moreover, HS can cause a 15–20% reduction in the fertilization rate of boar semen and a 10–15% decrease in the litter size of sows inseminated with HS-exposed boar semen and a 0.5–0.9 reduction in viable piglets per litter [8,9]. From a production perspective, reduced sperm quality directly impairs the efficiency of artificial insemination. Heat-induced sperm DNA damage not only decreases fertilization capacity but also elevates the risk of early embryonic loss, ultimately lowering the farrowing rate and litter size of bred sows [10,11,12]. These negative impacts severely restrict the reproductive efficiency and production benefits of the pig industry. Currently, mitigation measures such as cooling systems in boar houses and dietary supplementation with L-arginine or multiple antioxidants have limited effects [13,14], highlighting an urgent need for the development of more efficient intervention strategies.

In the mechanism of HS-induced damage to the male reproductive system, SCs, the only somatic cells in the testis that directly interact with developing germ cells, become the primary target. HS (exposure to 43 °C for 30 min or longer) can cause structural damage to SCs, including nuclear shrinkage, lipid droplet accumulation, swelling of the smooth endoplasmic reticulum, and mitochondrial degeneration. Meanwhile, it inhibits the expression of key functional proteins such as transferrin and WT1, thereby reducing cell viability and proliferative capacity [15,16,17]. More importantly, HS disrupts the integrity of the blood–testis barrier (BTB) [18,19,20] by downregulating the expression of tight junction-related proteins such as Occludin, Zonula Occludens-1 (ZO-1), Claudin-11, and the androgen receptor (AR), increasing barrier permeability and triggering the production of anti-sperm antibodies and autoimmune reactions [21,22,23]. At the molecular level, HS can activate apoptotic signaling pathways, leading to the upregulation of apoptotic-related proteins Caspase-3/8/9 and Bax, and the downregulation of Bcl-2. It also induces oxidative stress, characterized by decreased total antioxidant capacity (T-AOC) and superoxide dismutase (SOD) activity, and increased malondialdehyde (MDA) and reactive oxygen species (ROS) content. Additionally, HS results in decreased mitochondrial membrane potential (MMP), imbalanced mitochondrial dynamics (decreased Mfn1/2 expression and increased Drp1 expression), release of cytochrome C (Cyt C), and ATP depletion, ultimately inducing SC apoptosis [24].

PTD-FNK is an artificially engineered anti-apoptotic fusion protein. It is produced by IPTG-induced prokaryotic expression in Escherichia coli and subsequently subjected to refolding, affinity and purification, composed of FNK (a mutant derived from the crystal structure of rat Bcl-xL) and the protein transduction domain PTD (YGRKKRRQRRR) of HIV/TAT [25]. As the only gain-of-function mutant among mammalian anti-apoptotic factors, FNK exhibits superior anti-apoptotic activity to Bcl-xL and can enhance cellular resistance to various stimuli [26]. In vitro, PTD-FNK rapidly transduces into cells within one hour and localizes at mitochondria, maintaining cytoprotective effects for more than 12 h. In vivo, systemic administration allows wide tissue distribution; the maximal tissue concentration occurs at 3 h post-injection with a half-life of ~3.5 h, and the protein is eliminated mainly through intracellular proteolysis without significant toxicity at effective doses [25,27]. PTD-FNK has demonstrated significant cell-protective application value in multiple fields: In neuroprotection, when injected into mice, it can be delivered to various tissues including the brain, localize to mitochondria, and prevent delayed death of hippocampal neurons induced by transient global ischemia [25]. It also protects neurons from Fas-, nitric oxide-, or freeze–thaw-induced cell death [26,28,29]. In guinea pigs, TAT-FNK can alleviate kanamycin-induced death of cochlear sensory hair cells by inhibiting Caspase-9 activation in vitro [30,31]. In the field of reproduction, adding 300 nmol/L or 10 nmol/L PTD-FNK to the freezing preservation diluent of boar semen can enhance the in vitro fertilization capacity of post-thawed sperm or reduce the apoptosis rate by maintaining the integrity of sperm mitochondria and DNA, as well as inhibiting the activity or down-regulating the expression of Caspase-3 and Caspase-9, respectively. Moreover, the 10 nmol/L dose can reduce the dosage and suggest that the protective effect is related to the mitochondrial intrinsic pathway [32]. In respiratory system protection, both pre-treatment and post-treatment with PTD-FNK in rats can inhibit Caspase-3 activation and exert anti-apoptotic effects on alveolar epithelial cells and capillary endothelial cells [33]. Given its strong anti-apoptotic and anti-oxidative functions, we utilized PTD-FNK to investigate its protective effect against heat stress injury in boar SCs.

Given the core role of SCs in maintaining male reproductive function [34] and the efficient anti-apoptotic and cell-protective properties of PTD-FNK [35,36], this study aimed to systematically explore the protective effect and molecular mechanism of PTD-FNK against HS-induced damage in boar SCs. Firstly, SCs were isolated and cultured from the testes of 2-week-old boars [37], and cell purity was identified by hematoxylin-eosin (HE) staining, Oil Red O (ORO) staining, and GATA4 immunofluorescence. Subsequently, an HS model (43 °C for 1 h) was established based on cell viability and heat shock protein HSP70 expression, and 0.1 nM was selected as the optimal concentration of PTD-FNK. The protective effect of PTD-FNK against HS-induced damage was clarified by detecting the expression of BTB-related proteins, oxidative stress indicators, apoptotic-related molecules, and mitochondrial function parameters. Furthermore, combined with transcriptome sequencing and qRT-PCR validation, key differentially expressed genes such as *ID3*, *H2AX*, *CDKN1C*, *TP53*, *DDIT3*, and *RAD51* were screened, and pathway dependence was verified using the PI3K inhibitor LY294002. Ultimately, this study confirmed that PTD-FNK alleviates HS-induced apoptosis of boar SCs through activating the PI3K/AKT signaling pathway and multi-target regulation of the integrity of the BTB, the balance of oxidative stress, mitochondrial dynamics, and the expression of apoptosis-related genes, providing a new theoretical basis and technical strategy for improving the reproductive performance of boars under high-temperature environments.

## 2. Materials and Methods

### 2.1. Isolation and Culture of SCs

Experimental boars were obtained from pig farms in the Guangxi Zhuang Autonomous Region and raised under standardized feeding and management conditions. Castration was performed on 2-week-old healthy boars by standardized veterinary procedures to collect testes, and routine anti-infection treatment was given postoperatively without the use of analgesics. No euthanasia was performed throughout the experiment, and all animals were returned to routine feeding until marketing after the experiment. After the piglets were restrained, local anesthesia was administered with 2% lidocaine (0.5 mL per testis). An incision was made at the midline of the scrotum, extending 2–3 cm downward. The testes and spermatic cords were exposed through blunt dissection, and the testes were excised after double ligation of the spermatic cords. The excised testes were immediately immersed in ice-cold PBS supplemented with 5× penicillin-streptomycin (PS). Primary porcine SCs were isolated using a two-step enzymatic digestion method [37]. The specimens were kept at 4 °C for subsequent isolation of SCs. After removing the tunica albuginea, the testicular tissue was disinfected and minced before being treated with 2 mg/mL collagenase IV at 37 °C for 30 min. Cells were collected by centrifugation at 1000 r/min for 5 min. The resulting pellet was washed twice with PBS and subsequently treated with 5 mg/mL DNase I at 37 °C for 20 min. The reaction was terminated by adding complete culture medium. The mixture was filtered through 200 and 400 mesh sieves. Then the filtered fluid was centrifuged at 1000 r/min for 5 min. The pellet was washed twice with PBS before being treated with red cell lysis buffer at room temperature for 5 min to eliminate erythrocytes, and the treated pellet was washed three more times with PBS. Isolated cells were cultured in T25 flasks using DMEM medium containing 10% fetal bovine serum (FBS) and 1% PS at 37 °C with 5% CO_2_. All experiments in this study were performed using P1 primary SCs. The seeding density was set at 2 × 10^6^ cells/cm^2^ for all experimental groups. Experimental groups: The cells were treated as follows, control group(CON), the HS group was treated at 43 °C for 1 h (HS), the LY294002 alone group (LY) for 2 h, the HS-stimulated followed by LY294002 treatment group (HS + LY) for 2 h, PTD-FNK pretreatment for 1 h followed by HS group (HS + PTD-FNK); PTD-FNK pretreatment for 1 h plus HS for 1 h followed by LY294002 treatment group (HS + PTD-FNK + LY). N ≥ 3. The measured indicators included cell apoptosis, oxidative stress levels, the expression of tight junction-related genes, and changes in the PI3K/AKT signaling pathway.

PTD-FNK fusion protein was chemically synthesized by Shenggong Biotech (Shanghai) Co., Ltd. (Shanghai, China), and its purity was confirmed to exceed 95%. Lyophilized PTD-FNK powder was reconstituted using sterile PBS (pH 7.4). The stock solution was sterilized via a 0.22 μm filter, divided into single-use aliquots, and stored at −80 °C to avoid repeated freeze–thawing. Working solutions were freshly diluted in complete culture medium immediately prior to cell treatment.

All animal procedures in this study were approved by the Animal Experimental Ethics Committee of Guangxi University (No. GXU-2022-211, issued on 10 March 2022) and carried out in strict compliance with relevant animal ethical regulations and the ARRIVE guidelines.

### 2.2. HE Staining

Once SCs on slides reached 80% confluence, they were stained using the HE staining kit (G1120, Solarbio, Beijing, China) per the manufacturer’s protocol. After being washed three times with PBS for 5 min each to clean residual medium and debris, the cells were fixed with 4% paraformaldehyde for 15 min and thoroughly rinsed with PBS. They were then stained with hematoxylin for 2 min before being differentiated in 1% hydrochloric acid alcohol for 10 s. The cells were stained with eosin for 2 min, followed by a distilled water rinse. After being air-dried, the cells were mounted with neutral gum for microscopic observation of cellular morphology.

### 2.3. Oil Red O (ORO) Staining of SCs

SCs on the slides were stained following the manufacturer’s instructions of the ORO staining kit (G1262, Solarbio, Beijing, China). Briefly, the cells were fixed with ORO fixative at room temperature for 30 min after being washed three times with PBS. Subsequently, the cells were washed twice with distilled water and then immersed in 60% isopropanol for 5 min to remove excess fixative. Next, the cells were stained with ORO stain solution for 20 min, followed by extensive washing with distilled water. The nucleus was counterstained with Mayer’s hematoxylin for 1 min. The cells were air-dried after being immersed in ORO buffer for 1 min to remove excess liquid.

### 2.4. Immunofluorescence Identification of SCs

For the immunofluorescence assay, SCs on the slides at a confluence of 80% were fixed with 4% paraformaldehyde for 15 min, then they were washed three times with PBS. Subsequently, the cells were treated with 0.1% Triton X-100 for 10 min for permeabilization. The cells were then blocked with 5% goat serum for 30 min. For immunofluorescence staining, the cells were incubated with GATA4 antibody overnight at 4 °C or for 1 h at room temperature, followed by incubation with FITC-conjugated secondary antibody after intensive washing with PBS. The nuclei were stained with DAPI. Images were obtained with a fluorescence microscope.

### 2.5. Viability Assay of SCs

Cell viability was evaluated by the CCK-8 kit (CA1210, Solarbio, Beijing, China). SCs were seeded into 96-well plates at 10^4^ cells/mL. For the temperature viability study, the cells were cultured at 37 °C, 39 °C, 41 °C, 43 °C, and 47 °C. For PTD-FNK effect assessment, the cells were exposed to 0, 0.01, 0.1, 1, 10, and 100 nM PTD-FNK at 43 °C. For LY294002 impact analysis, the cells were treated with 0, 1, 2, 5, 10, and 20 μM LY294002 at 43 °C. At set times, medium was replaced with 10% CCK-8 solution. After 2 h incubation at 37 °C, absorbance was read at 450 nm via a microplate reader.

### 2.6. Determination of ATP and Cyt C Levels Using ELISA

Intracellular ATP and Cyt C levels were measured via ELISA kits (MEIMIAN, Yancheng, China). The cells were collected per the instructions, with the supernatant retained for analysis. The ELISA procedure followed the manufacturer’s guidelines: setting up standard, blank, and test wells; adding enzyme-labeled reagent and incubating; washing; adding chromogen and stop solutions; and measuring OD values at 450 nm with the blank well as a control.

### 2.7. Determination of SCs Oxidative and Antioxidative Parameters

The levels of T-AOC, MDA, SOD activity, and ROS in SCs were measured per instructions (Solarbio, Beijing, China). For T-AOC detection, SCs were lysed by adding 1 mL of pre-cooled extraction buffer and sonicated (200 W, 3 s on/10 s off, 30 cycles). The lysate was spun at 1200 r/min for 5 min at 4 °C, and the liquid was collected. The supernatant was mixed with the detection reagent, and the mixture was reacted for 10 min, followed by measurement of the OD at 593 nm. For MDA detection, SCs were lysed via sonication following centrifugation to eliminate the supernatant. The lysate was centrifuged at 8000 r/min for 10 min at 4 °C. The supernatant was then incubated at 95 °C for 60 min before being centrifuged at 10,000 r/min for 10 min after cooling. The OD value of 200 μL of the supernatant was measured using a microplate reader. For SOD activity detection, SCs were lysed using centrifugation at 8000 r/min for 10 min at 4 °C. The supernatant was incubated at 37 °C for 30 min, and the OD was measured at 560 nm. For ROS detection, SCs were washed twice with PBS before being resuspended in DCFH-DA solution, and then they were treated with a 50 μM hydrogen donor for induction. The cells were incubated at 37 °C for 30 min. After being washed, the supernatant was resuspended for fluorescence detection (excitation 500 nm, emission 520 nm). Negative controls were also set up.

### 2.8. Determination of SCs MMP and Intracellular Ca^2+^

The MMP and intracellular Ca^2+^ levels in SCs were quantified per manufacturer protocol (Solarbio, Beijing, China). For MMP measurement, a staining working solution was prepared. A positive control was set up by treating cells with CCCP. Following PBS washing, SCs were stained and incubated (37 °C, 20 min), then resuspended in the staining buffer after post-staining washes for flow cytometry analysis (excitation 488 nm, emission 530 nm). For Ca^2+^ quantification, the cells were loaded with Fluo-3 AM, triple-washed with HBSS, incubated (37 °C, 20 min), and fluorescence intensity was measured via a microplate reader (excitation 506 nm, emission 526 nm).

### 2.9. SCs Treatment with the PI3K Inhibitor LY294002

The cultures were established in 6-well plates using a complete medium to investigate the effects of HS and the PI3K inhibitor LY294002 on SCs. When the cell confluency reached 90%, LY294002 (2 μM) was administered to the cells. After 2 h of treatment, cells from each group were collected for total RNA extraction.

### 2.10. qRT-PCR Analysis of SCs Gene Expression

Gene expression analysis was conducted as follows: RNA isolated with Trizol (R411, Vazyme, Nanjing, China) was reverse transcribed to cDNA using All-In-One 5× RT Master Mix (R211, Vazyme, Nanjing, China), followed by qRT-PCR amplification with SYBR Master Mix (Q712, Vazyme, Nanjing, China) under cycling conditions: 95 °C/30 s; 30 cycles of 95 °C/5 s, 62 °C/30 s, 72 °C/30 s; final extension at 72 °C/5 min. Expression was calculated via the 2^−∆∆Ct^ method. All primers were synthesized by GenScript Biotechnology Corporation (Nanjing, China), and primer sequences were designed based on the NCBI Primer-Blast tool. GAPDH was used as the housekeeping gene for normalization. Primer information is detailed in Table 1.

### 2.11. Transcriptomic Sequencing of SCs

SCs were plated in 6-well plates and maintained until 80% confluency. Experimental groups included: the CON group, cultured at 37 °C for 1 h; the HS group, cultured at 43 °C for 1 h; and the HS + PTD-FNK group, PTD-FNK pretreatment for 1 h followed by 43 °C for 1 h. After the experimental treatments, the cells were harvested for RNA-seq (Majorbio, Shanghai, China) and data analysis via Majorbio Cloud Platform (Majorbio).

### 2.12. Western Blotting

Cells were harvested and lysed in RIPA reagent supplemented with protease inhibitors, phosphatase inhibitors, and PMSF. All protein samples were quantified using a BCA Protein Assay Kit (Beyotime, Shanghai, China) to ensure a consistent loading concentration of 30 μg per sample. The samples were incubated in protein loading buffer at 95 °C for 10 min for denaturation. Subsequently, proteins were separated by SDS-PAGE and transferred onto polyvinylidene difluoride (PVDF) membranes. The membranes were blocked with 5% non-fat milk for 1 h at room temperature, then incubated overnight at 4 °C with a primary antibody against Bax (1:1000; CST, Danvers, MA, USA), Bcl-2 (1:1000; HUABIO, Hangzhou, China), P-PI3K (1:1000; CST, Danvers, MA, USA), PI3K (1:1000; ABclonal, Wuhan, China), P-AKT (1:1000; ABclonal), AKT (1:1000; Abcam, Cambridge, UK), and actin (1:1000; CST, Danvers, MA, USA,). After extensive washing with TBST buffer, the membranes were incubated with a horseradish peroxidase (HRP)-conjugated secondary antibody (Beyotime, China) for 1 h at room temperature. Protein bands were visualized using an ECL chemiluminescence detection system, and the relative expression levels were quantified using ImageJ software (version 1.54f, National Institutes of Health, Bethesda, MD, USA).

### 2.13. Statistical Analysis

Statistical analyses were conducted with GraphPad Prism 10.2.2. Two-group comparisons were completed using unpaired *t*-tests, while multi-group analyses were finished using one-way ANOVA. Statistical significance was denoted as follows: ns for *p* > 0.05 (not significant), * for *p* < 0.05 (significant), ** for *p* < 0.01 (highly significant), *** for *p* < 0.001 (very significant), and **** for *p* < 0.0001 (extremely significant). The error bars in all graphs represent the standard error of the mean (SEM).

## 3. Results

### 3.1. Identification of SCs

**Figure 1:**
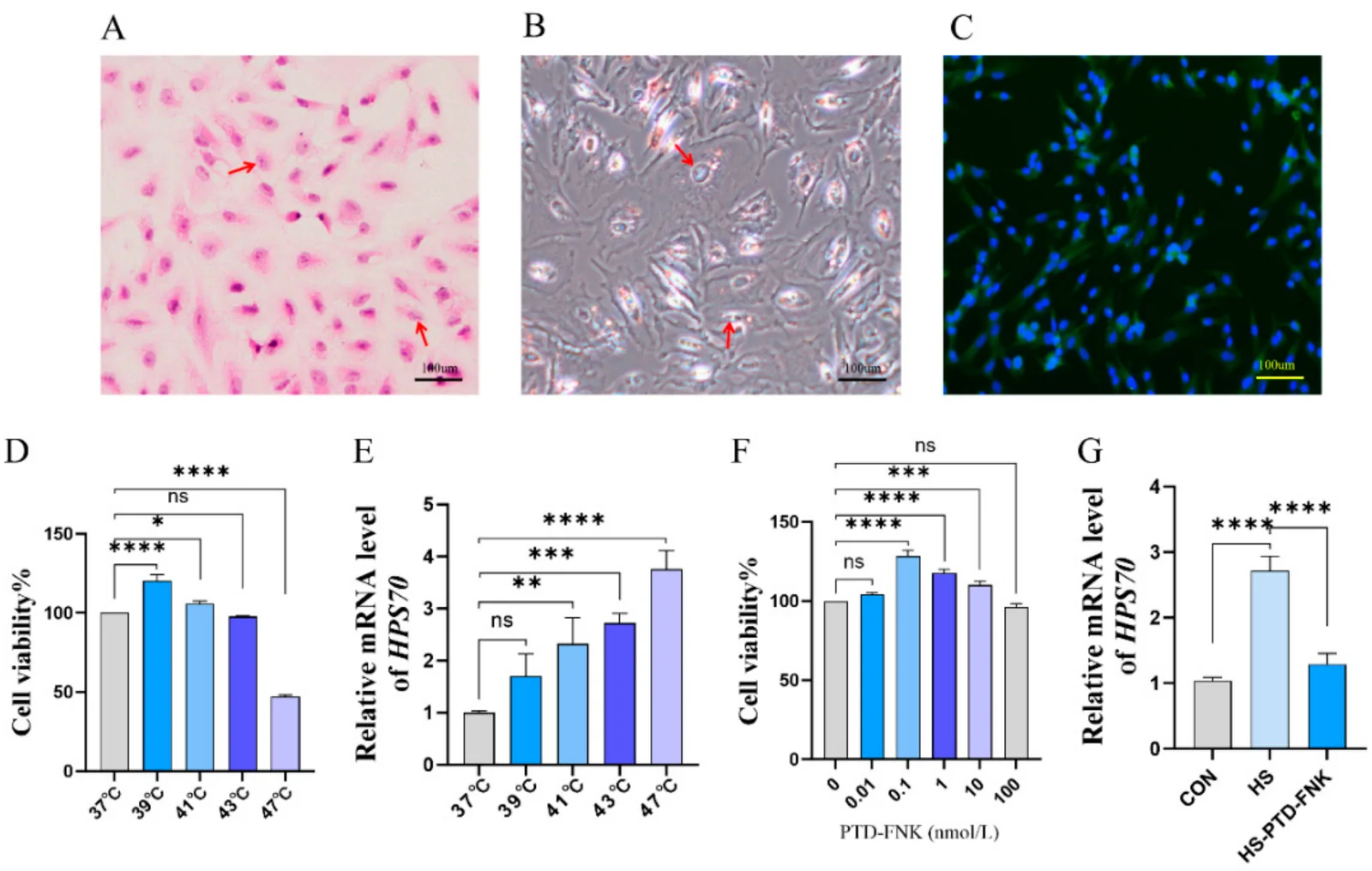
Establishment of the HS model. (**A**) HE staining of SCs, with arrows indicating the nucleolus within the cell nucleus; (**B**) ORO staining of SCs, with arrows indicating the bipolar bodies within the cell nucleus; (**C**) Immunofluorescence staining for *GATA4*, using primary antibody *GATA4*, followed by incubation with FITC-conjugated AffiniPure Rabbit Anti-Goat IgG (H + L). The cell nucleus is stained with DAPI (blue), and green fluorescence represents the expression level of *GATA4*; (**D**) SCs viability under different temperature (37 °C, 39 °C,41 °C,43 °C,47 °C); (**E**) *HSP70* expression under different temperature (37 °C, 39 °C, 41 °C, 43 °C, 47 °C); (**F**) SCs viability under 43 °C with different concentration of PTD-FNK (0, 0.01, 0.1, 1, 10 and 100 nM); (**G**) PTD-FNK modulates HSP70 expression in heat-stressed SCs (ns, not significant, * *p* < 0.05, ** *p* < 0.01, *** *p* < 0.001, and **** *p* < 0.0001).

Primary SCs were characterized via histochemical and immunofluorescence assays. HE staining revealed basophilic purple-red nuclei, whereas the cytoplasm displayed pale eosinophilic staining. Cells showed irregular morphology with multiple cytoplasmic protrusions; nuclei were oval or irregular, containing 2–3 distinct nucleoli (Figure 1A). ORO staining demonstrated numerous round lipid droplets of varying sizes stained bright red within the cytoplasm. Moreover, characteristic bipolar bodies of SCs could be observed in the nuclei (Figure 1B). Immunofluorescence analysis further detected prominent green fluorescence corresponding to the SC marker GATA4 (Figure 1C), confirming strong GATA4 expression in the isolated cells. Quantitative analysis from five random microscopic fields demonstrated that the proportion of GATA4-positive cells reached 93.65 ± 1.76%, confirming high purity of isolated primary porcine SCs. SCs were subjected to thermal gradients (37 °C, 39 °C, 41 °C, 43 °C, 47 °C; 1 h) with viability and HSP70 expression assessed to determine the optimal temperature for establishing an HS model. Viability peaked at 39 °C and 41 °C (*p* < 0.05) but sharply declined at 47 °C (*p* < 0.05; Figure 1D). HSP70 expression exhibited temperature-dependent upregulation (*p* < 0.05; Figure 1E). However, 47 °C-induced viability loss and apoptosis precluded its use for HS modeling, establishing 43 °C as the optimal condition. Dose optimization was performed to evaluate PTD-FNK-mediated rescue of HS-induced BTB damage. SCs pretreated with PTD-FNK (0.01, 0.1, 1, 10, and 100 nM) prior to HS exposure (43 °C, 1 h), compared to the CON group, exhibited enhanced viability at 0.1, 1 and 10 nM (*p* < 0.05), while 0.01 and 100 nM showed no significant effects (*p* > 0.05). An amount of 0.1 nM was established as the optimal dose for HS damage rescue (Figure 1F). The HS model exhibited markedly elevated HSP70 versus the CON (*p* < 0.05), confirming effective stress activation. PTD-FNK (0.1 nM) treatment reversed this response, achieving HSP70 restoration to baseline levels (*p* < 0.05; Figure 1G).

### 3.2. PTD-FNK Mitigates HS-Induced BTB Disruption and Oxidative Stress in SCs

**Figure 2:**
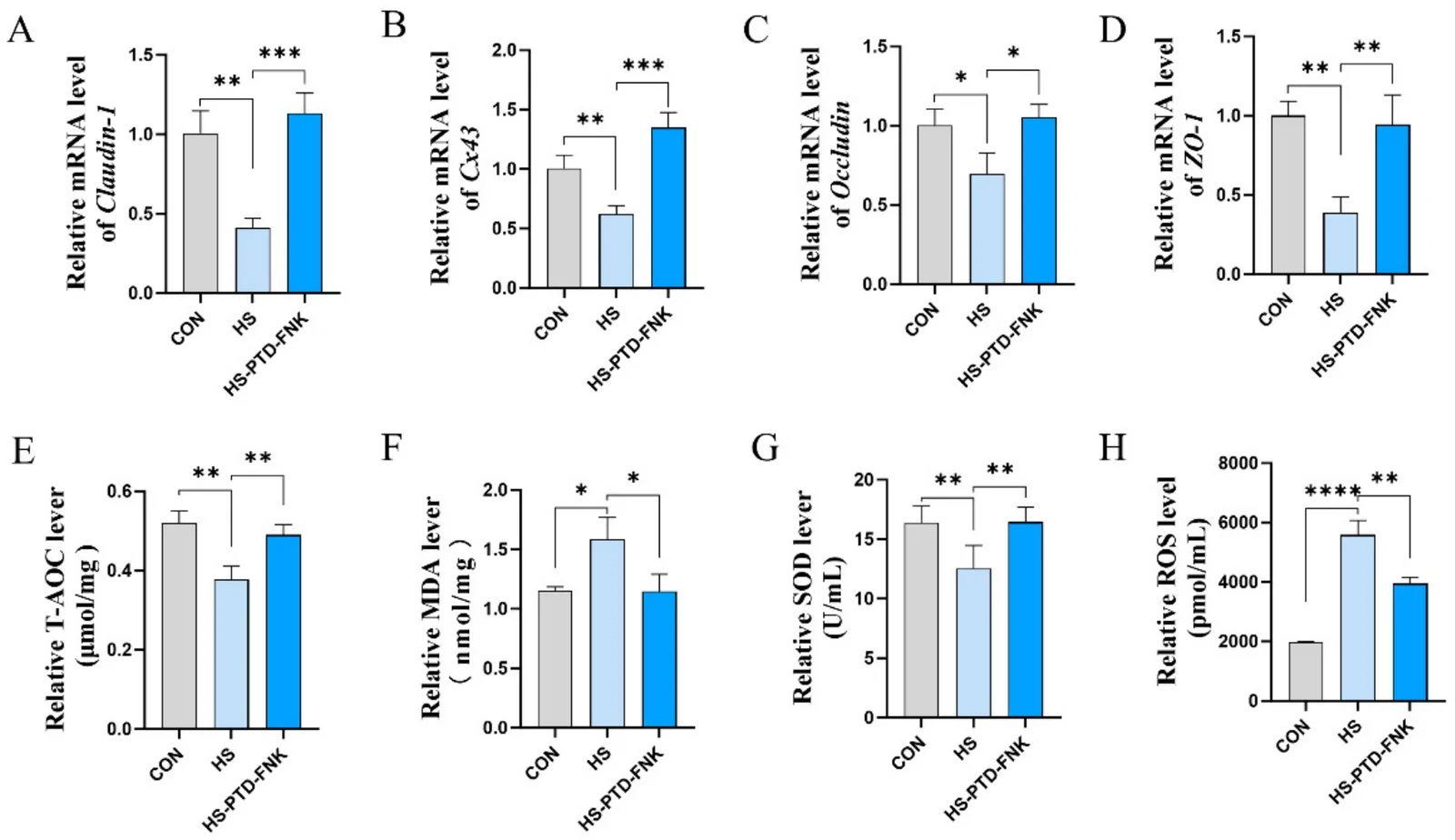
PTD-FNK Mitigates HS-Induced BTB Disruption and Oxidative Stress in SCs. Cells were pretreated with PTD-FNK for 1 h, followed by treatment at 43 °C for 1 h. To check the transcriptional levels of *Claudin-1* (**A**), *Occludin* (**B**), *ZO-1* (**C**), and *Cx43* (**D**) by qRT-PCR. To detect the contents of T-AOC (**E**), MDA (**F**), SOD (**G**) and ROS (**H**) (ns, not significant, * *p* < 0.05, ** *p* < 0.01, *** *p* < 0.001, and **** *p* < 0.0001).

HS downregulated BTB-associated tight junction proteins (Claudin-1, Occludin, ZO-1 and Cx43; *p* < 0.05), compromising barrier integrity. PTD-FNK (0.1 nM) rescued the expression of tight junction proteins to basal levels (*p* < 0.05), effectively reversing HS-induced BTB disruption (Figure 2A–D). PTD-FNK mitigated HS-induced oxidative stress in SCs, as evidenced by redox marker analysis. HS exposure markedly reduced T-AOC/SOD activity and elevated MDA/ROS content versus the control (*p* < 0.05), confirming oxidative injury. PTD-FNK treatment rescued T-AOC/SOD activity and attenuated MDA accumulation to baseline (*p* < 0.05), though ROS levels remained elevated post-treatment (Figure 2E–H). From these data, it was suggested that PTD-FNK alleviates oxidative damage through T-AOC/SOD stabilization and MDA suppression.

### 3.3. PTD-FNK Mitigates HS-Induced Apoptosis and Modulates Mitochondrial Dynamics in SCs

**Figure 3:**
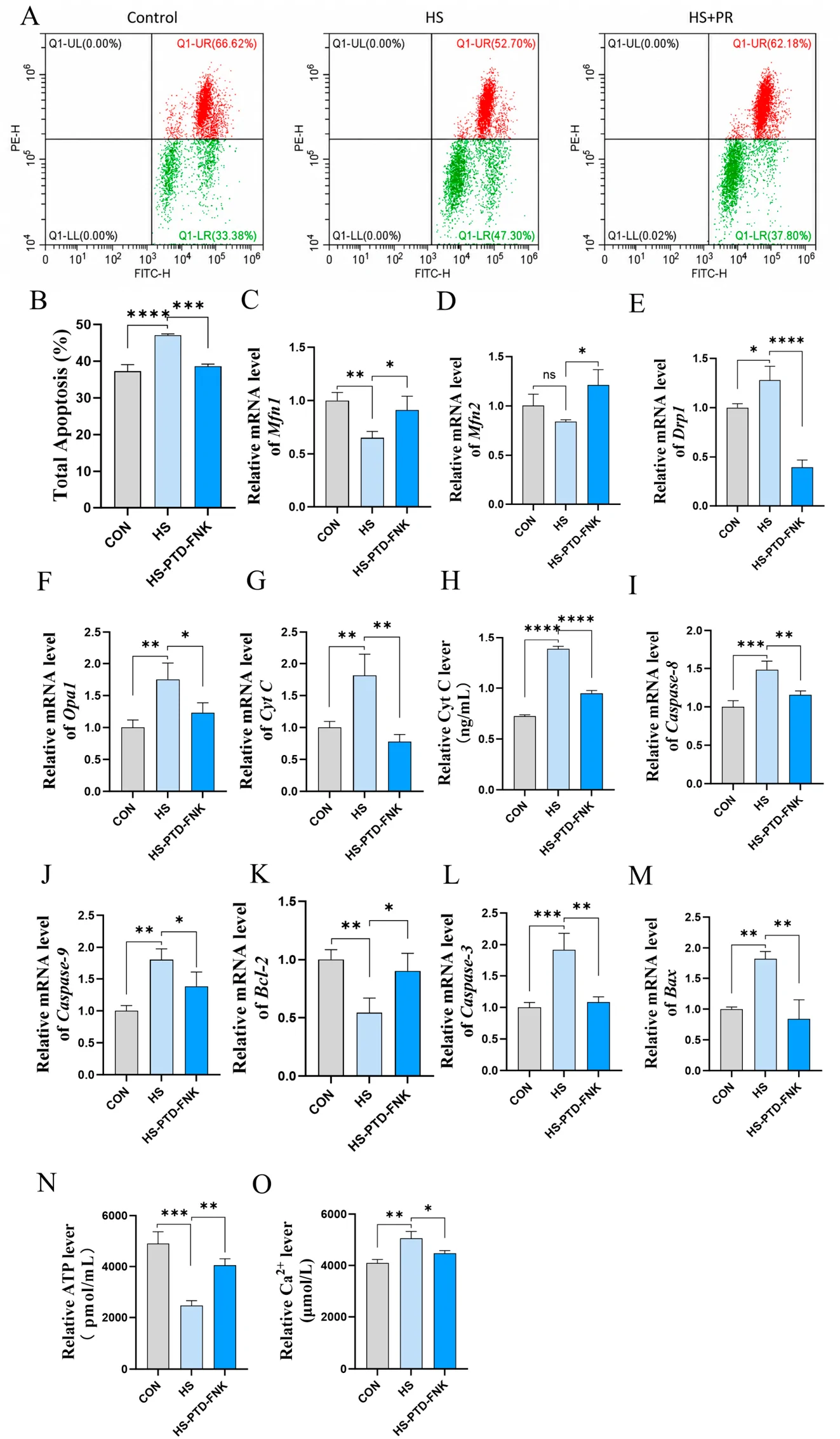
PTD-FNK Regulates HS-Driven Mitochondrial Dynamics and Mitigates Apoptosis in SCs. Cells were pretreated with PTD-FNK for 1 h, followed by treatment at 43 °C for 1 h. (**A**) Flow Cytometric Assessment of MMP; (**B**) Apoptotic rate. To check the transcriptional levels of *Mfn1* (**C**), *Mfn2* (**D**), *Drp1* (**E**), *Opa1* (**F**), *Cyt C* (**G**), *Caspase-8* (**I**), *Caspase-9* (**J**), *Bcl-2* (**K**), *Caspase-3* (**L**) and *Bax* (**M**) by qRT-PCR. To detect the contents of Cyt C content (**H**), ATP content (**N**) and Ca^2+^ content (**O**) (ns, not significant, * *p* < 0.05, ** *p* < 0.01, *** *p* < 0.001, and **** *p* < 0.0001).

MMP was analyzed by flow cytometry. Compared to the CON, HS induced MMP depolarization (green fluorescence, monomeric mitochondria), correlating with elevated apoptosis rates (*p* < 0.05; Figure 3A,B). PTD-FNK restored MMP hyperpolarization (red fluorescence, aggregated mitochondria), normalizing apoptosis to baseline levels (*p* < 0.05). HS led to suppressed *Mfn1* (*p* < 0.05) and elevated *Drp1*, *Opa1*, and Cyt C (*p* < 0.05), with Mfn2 trending downward (*p* > 0.05). PTD-FNK reversed this imbalance, upregulating Mfn1/2 and downregulating fission/apoptotic markers (*Drp1*, *Opa1*, *Cyt C*; *p* < 0.05; Figure 3C–H). Cyt C protein levels mirrored transcriptional changes. Collectively, PTD-FNK rescues HS-induced mitochondrial dysfunction by coordinating fusion–fission dynamics and suppressing apoptogenic signaling. HS triggered apoptotic signaling in SCs, upregulating pro-apoptotic markers (*Bax*, *Caspase-3/8/9*; *p* < 0.05) while suppressing *Bcl-2* (*p* < 0.05; Figure 3I–M). PTD-FNK reversed this trend, rescuing *Bcl-2* expression (*p* < 0.05). Concurrently, HS induced Ca^2+^ overload and ATP depletion (*p* < 0.05), which were normalized by PTD-FNK through Ca^2+^ flux reduction and ATP restoration (*p* < 0.05; Figure 3N,O). These findings demonstrate PTD-FNK maintains cellular homeostasis via modulation of apoptotic mechanisms and bioenergetic recovery.

### 3.4. PTD-FNK Modulates HS-Induced Transcriptomic Changes in SCs Validated Using qRT-PCR

**Figure 4:**
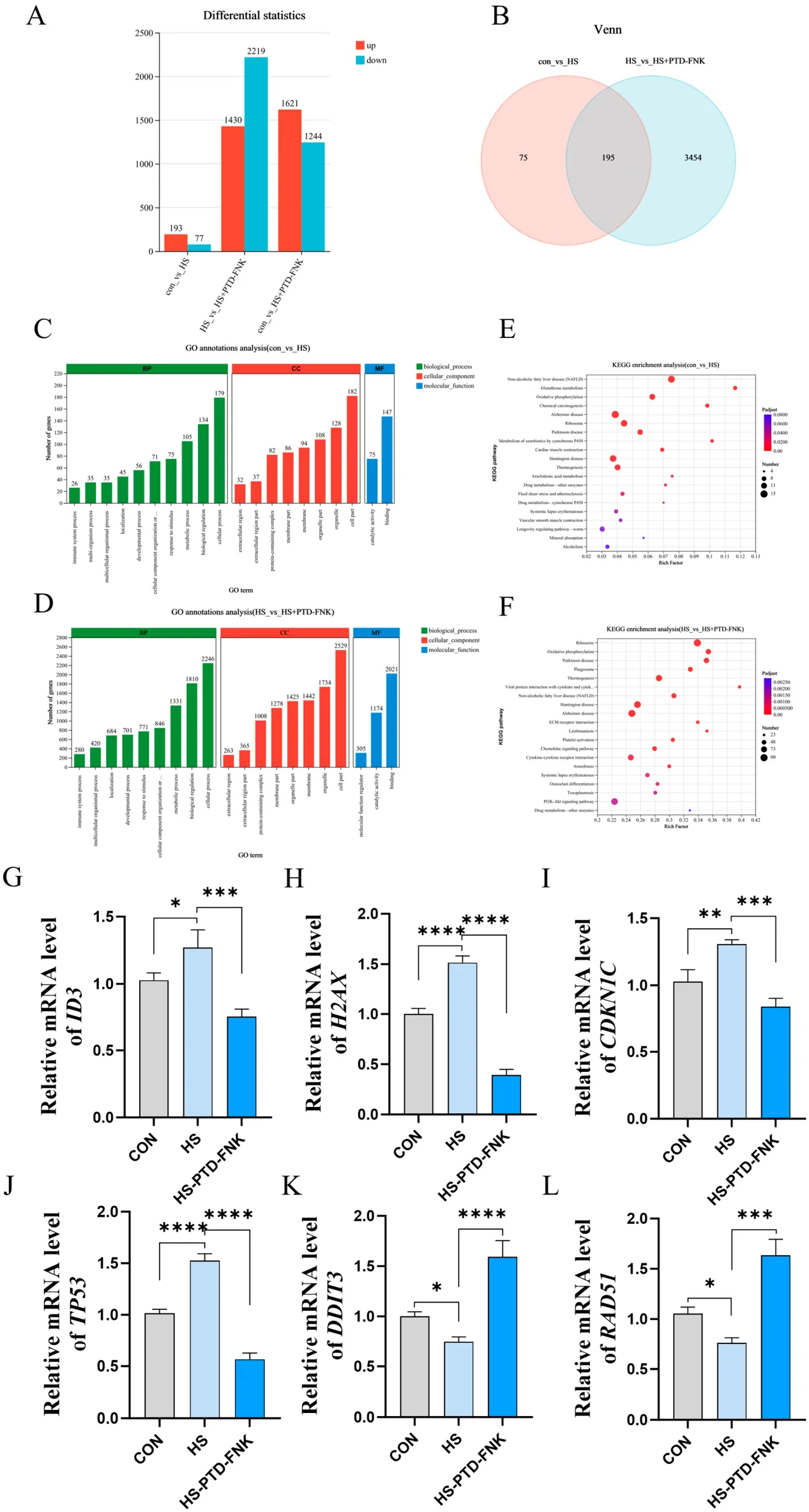
Transcriptomic analysis of HS and PTD-FNK treatment in SCs and qRT-PCR validation of DEGs. Cells were pretreated with PTD-FNK for 1 h, followed by treatment at 43 °C for 1 h. (**A**) Differential expression analysis between the CON, HS, and HS + PTD-FNK groups; (**B**) Venn diagrams of the gene sets for the CON vs. HS and HS vs. HS + PTD-FNK; (**C**,**D**) GO/KEGG enrichment analysis in the CON vs. HS; (**E**,**F**) GO/KEGG enrichment analysis in HS vs. HS + PTD-FNK. To check the transcriptional levels of *ID3* (**G**); *H2AX* (**H**); *CDKN1C* (**I**); *TP53* (**J**); *DDIT3* (**K**); *RAD51* (**L**) by qRT-PCR. (ns, not significant, * *p* < 0.05, ** *p* < 0.01, *** *p* < 0.001, and **** *p* < 0.0001).

Using transcriptomic profiling, 270 DEGs (|log2FC| ≥ 1, *p* < 0.05) in HS vs. CON (193 up/77 down) were identified, and 3649 DEGs in HS + PTD-FNK vs. HS (1430 up/2219 down), with 195 overlapping genes (Figure 4A,B). GO annotation analysis of the above DEGs was carried out. The 270 DEGs in the CON vs. HS group were primarily enriched in binding (147 genes) and catalytic activity (75 genes) within molecular functions. In cellular components, DEGs were mainly involved in cell parts (128 genes), organelles (108 genes), and organelle parts (94 genes); in biological processes, DEGs were predominantly associated with cellular processes (197 genes), biological regulation (134 genes), and metabolic processes (105 genes) (Figure 4C). The 3649 DEGs in the HS vs. HS + PTD-FNK group were enriched in binding (2021 genes) and catalytic activity (1174 genes) within molecular functions. In cellular components, DEGs were mainly involved in cell parts (2529 genes), organelles (1734 genes), and cell membrane (1442 genes); in biological processes, DEGs were predominantly associated with cellular processes (2246 genes), biological regulation (1810 genes), and metabolic processes (1131 genes) (Figure 4D).

KEGG enrichment analysis of the above DEGs was conducted. The DEGs in the CON vs. HS group were mainly enriched in signaling pathways related to apoptosis, redox processes, and mitochondrial metabolism (glutathione metabolism, oxidative phosphorylation, etc.), the PI3K-AKT signaling pathway, and the MAPK signaling pathway, with the majority of genes being upregulated (Figure 4E). The DEGs in the HS vs. HS + PTD-FNK group were similarly enriched in apoptosis and mitochondrial metabolism-related pathways, such as the PI3K-AKT signaling pathway, MAPK signaling pathway, and cell adhesion pathways, with the majority of genes being downregulated and a small number of genes upregulated (Figure 4F).

Transcriptome sequencing data validation targeted six PTD-FNK-modulated genes (*ID3*, *H2AX*, *CDKN1C*, *TP53*, *DDIT3*, *RAD51*) linked to proliferation/DNA damage. HS upregulated *ID3*, *H2AX*, *CDKN1C*, and *TP53* (*p* < 0.05; Figure 4G–J), which were rescued by PTD-FNK (*p* < 0.05). Conversely, HS suppressed *DDIT3* and *RAD51* (*p* < 0.05; Figure 4K,L); similarly, PTD-FNK restored their expression (*p* < 0.05). qRT-PCR validation corroborated transcriptome sequencing data reliability.

### 3.5. PTD-FNK Alleviated HS-Induced Apoptosis of SCs via the PI3K/AKT Pathway

**Figure 5:**
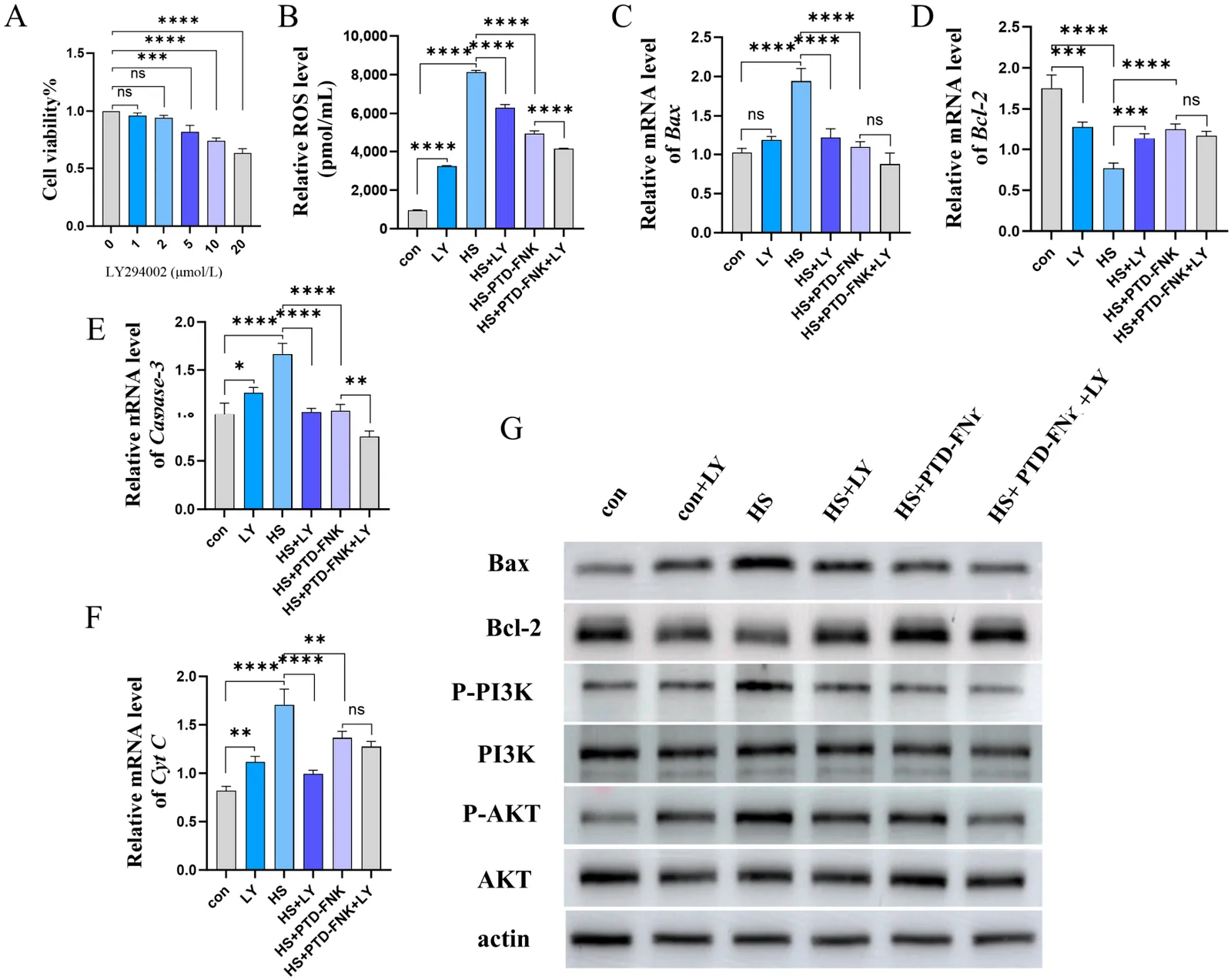
Effects of LY294002 and PTD-FNK on HS-Induced SCs. (**A**) LY294002 Dose-Dependent Viability Profiling (0, 1, 2, 5, 10 and 20 μM); (**B**) Effects of LY294002 on ROS levels in HS-induced SCs; LY294002 Modulates Apoptotic Mediators: *Bax* (**C**), *Bcl-2* (**D**), *Caspase-3* (**E**), and *Cyt C* (**F**); (**G**) Effect of LY294002 on Bax, Bcl-2, p-PI3K, PI3K, p-Akt and Akt protein expression in HS-Induced SCs. (ns, not significant, * *p* < 0.05, ** *p* < 0.01, *** *p* < 0.001, and **** *p* < 0.0001) (the original Western blot pictures can be found in File S1).

To clarify whether HS induces SC damage via the PI3K/AKT pathway, the CCK-8 assay was employed to evaluate the effects of different concentrations of LY294002 (0, 1, 2, 5, 10, and 20 μM) on SC viability. The results showed that 2 μM LY294002 had no obvious cytotoxicity to SCs, while concentrations > 5 μM significantly inhibited SCs survival (*p* < 0.05; Figure 5A). Therefore, 2 μM LY294002 was selected for subsequent experiments to explore its regulatory role in the protective effect of PTD-FNK against HS-induced SCs damage.

qRT-PCR results revealed that LY294002 and PTD-FNK treatment alone significantly reduced ROS levels in the CON group (*p* < 0.05), whereas combined treatment with PTD-FNK remarkably reduced ROS content in the HS group (*p* < 0.05; Figure 5B). At the molecular level, LY294002 and PTD-FNK monotherapy downregulated the expression of pro-apoptotic markers (*Bax*, *Caspase-3*, and *Cyt C*) and upregulated the anti-apoptotic marker *Bcl-2* in SCs (*p* < 0.05). Compared with the HS + PTD-FNK group, the HS + PTD-FNK + LY group showed no significant differences in the expression levels of *Bax*, *Bcl-2*, and *Cyt C*, but exhibited a further downregulation of *Caspase-3* (*p* < 0.05; Figure 5C–F).

Further in-depth verification was performed at the protein level. Compared with the CON group, the expression levels of Bcl-2, p-PI3K and p-Akt were significantly decreased, while the expression of Bax was significantly increased in the HS model group. Compared with the HS model group, the expression levels of Bcl-2, p-PI3K and p-Akt were significantly up-regulated, and Bax expression was significantly down-regulated in the HS + PTD-FNK group. In addition, the expression of Bax/Bcl-2 was further significantly increased in the HS + PTD-FNK + LY group compared with the CON + LY group (Figure 5G).

Collectively, PTD-FNK exerts a protective effect against heat stress-induced damage by up-regulating the expression of p-PI3K and p-Akt and reducing the Bax/Bcl-2 ratio, thereby alleviating apoptosis of SCs caused by HS. This protective effect not only confirms the involvement of the PI3K/AKT signaling pathway in HS-mediated SC injury, but also demonstrates that PTD-FNK exerts its anti-apoptotic and antioxidant functions in a PI3K/AKT-dependent manner. These findings provide strong evidence that PTD-FNK protects against HS-induced SC injury through a PI3K/AKT-dependent mechanism.

## 4. Discussion

HS acts as a vital environmental stressor impairing boar reproductive performance under intensive production systems. HS disrupts the structural and functional integrity of SCs, triggers oxidative stress and cellular apoptosis, and damages the BTB, ultimately leading to reproductive dysfunction [3,15,16,17]. The fusion protein PTD-FNK is an artificially synthesized anti-apoptotic molecule. Existing studies have verified its potent cytoprotective effects in multiple models, including neuroprotection and semen cryopreservation [26,29].

Nevertheless, whether PTD-FNK can alleviate HS-induced SC injury and its underlying molecular mechanism remain largely unexplored. Via systematic in vitro experiments, the present study confirmed that pretreatment with an optimal concentration (0.1 nM) of PTD-FNK reverses HS-evoked SC damage through multi-target regulation, and its core protective mechanism depends on activation of the PI3K/AKT signaling pathway. These findings provide novel theoretical evidence and a potential molecular target for mitigating HS-related reproductive impairment in boars.

Consistent with multiple previous reports focused on porcine and rodent SCs, our results demonstrated that HS markedly reduces T-AOC and SOD activity, while elevating ROS and MDA levels in SCs [38,39]. Excess ROS further initiates apoptosis through coordinated multiple cascades: ROS accumulation disrupts MMP, triggers mitochondrial dysfunction, provokes intracellular Ca^2+^ overload and ATP exhaustion, and remodels the transcription of apoptosis-associated genes, collectively activating apoptotic signaling [40,41]. Despite this consensus, several in vivo investigations revealed that short-term mild HS only induces transient ROS elevation without persistent suppression of antioxidant enzyme activity in intact testicular tissue [42,43]. Such discrepancies are plausibly attributed to experimental differences: heat exposure temperature and duration, in vitro purified SC culture versus complex in vivo testicular microenvironment containing multiple cell populations, animal age, and sampling time after thermal stimulation. Isolated primary SCs lack paracrine support from germ cells and Leydig cells, rendering them more susceptible to continuous hyperthermia than testicular tissue in vivo. PTD-FNK pretreatment effectively reversed all HS-induced adverse alterations described above. PTD-FNK restored cellular redox equilibrium, stabilized MMP, balanced the expression of mitochondrial dynamics proteins Mfn1/Drp1, inhibited cytochrome C release, and modulated transcription of key genes including the Bax/Bcl-2/Caspase family, ID3, H2AX, CDKN1C, TP53, RAD51 and DDIT3. These observations expand the known antioxidant and anti-apoptotic functions of PTD-FNK, and confirm that this fusion protein synchronously regulates oxidative stress homeostasis, mitochondrial dynamics and DNA damage repair in SCs, reflecting its unique advantage of multi-target synergistic protection. In contrast, classical single-component antioxidants such as vitamin C mainly function via direct ROS scavenging to relieve oxidative damage, lacking coordinated regulatory effects on mitochondrial function, DNA repair and intercellular junction integrity [14,44,45].

As an indispensable physical barrier maintaining the homeostasis of the spermatogenic microenvironment, intact BTB relies on stable expression of tight junction proteins including *Claudin-1*, *Occludin*, *ZO-1*, and *Cx43* [6,20,21,22,23,46,47]. Our study verified that HS significantly downregulated these tight junction proteins, whereas PTD-FNK pretreatment effectively restores their expression and sustains structural and functional integrity of the BTB, representing a key innovation of this research. Previous work reported that *Lycium barbarum* polysaccharide protects BTB integrity via androgen receptor-dependent AKT phosphorylation [48,49,50]. Distinct from this single-axis regulatory mode, the BTB-protective effect of PTD-FNK is a downstream event of its broad-spectrum multi-target regulation, further highlighting its high efficiency and wide-spectrum protective capacity in the male reproductive system. Transcriptomic profiling revealed that differentially expressed genes (DEGs) between the control and HS groups were significantly enriched in the PI3K/AKT signaling pathway, and PTD-FNK treatment substantially remodeled the expression of pathway-related genes. Subsequent protein detection confirmed that HS suppressed the phosphorylation of PI3K and AKT and reduced Bcl-2 abundance, accompanied by upregulated Bax expression; PTD-FNK reversed these HS-induced changes. Further verification using the PI3K-specific inhibitor LY294002 demonstrated that the cytoprotective effects of PTD-FNK were partially abolished upon PI3K/AKT blockade. Combined intervention of PTD-FNK and LY294002 enhanced anti-apoptotic capacity by inhibiting Caspase-3 activation and restoring ROS homeostasis. Collectively, these data support the hypothesis that the protective function of PTD-FNK against HS injury relies largely on PI3K/AKT pathway activation. This mechanistic pattern echoes earlier findings showing that PTD-FNK suppresses apoptosis via PI3K/AKT signaling in acute lung injury [33]. However, the present study is the first to link this pathway to the regulation of oxidative stress, BTB integrity, and mitochondrial function in SCs, improving the molecular regulatory network of PTD-FNK in reproductive cell protection. Meanwhile, the key DEGs of *ID3*, *H2AX*, *CDKN1C*, *TP53*, *RAD51*, and *DDIT3* identified in this study provide important references for subsequent analysis of molecular markers of HS-induced damage.

## 5. Conclusions

In conclusion, PTD-FNK exerts a multi-target protective effect on alleviating oxidative stress, protecting mitochondrial function, and inhibiting apoptosis, which is mediated through the PI3K/AKT signaling pathway. This study provides a novel theoretical basis and technical strategy for improving the reproductive performance of boars in high-temperature environments, and lays a foundation for the clinical application of PTD-FNK in animal husbandry.

## Figures and Tables

**Table 1:** Primers of SCs differentially expressed genes.

Primer Name	Base Sequence (5′ to 3′)	
*ID3*	F:GGTGTGCTGCCTATCGGAAC	R:CCAGGATGTAGTCGATGACGC
*DDIT3*	F:GAAGAGGAAGAATCAAAAACCTTCA	R:GCCCTGGCTCCTCTGTCA
*CDKN1C*	F:TCGCTGGGACTACAACTTCC	R:AGGCCGTCGACAGACTCATC
*H2AX*	F:GGTGCTGGAGTATCTGACCG	R:GTTGAGCTCTTCGTCGTTGC
*TP53*	F:TACCACCATCCACTACAACTTCAT	R:CTCCCAGGACAGGCACAAA
*RAD51*	F:CTTCGGTGGAAGAGGAGAGC	R:CGGTGTGGAATCCAGCTTCT
*Caspase-3*	F:GCAGTTTTATTTGCGTGCTTC	R:TCCGTCTCAATCCCACAGTC
*Caspase-8*	F:CCAGGATTTGCCTCCGGTTA	R:TGTGGGATGTAGTCCAGGCT
*Caspase-9*	F:GACCTCCATCCTGTGGTGAT	R:GCATTTCCCTTGGCTCTGT
*Bax*	F:GGCCTCCTCTCCTACTTTGG	R:CTCAGCCCATCTTCTTCCAG
*Bcl-2*	F:AGGGCATTCAGTGACCTGAC	R:CGATCCGACTCACCAATACC
*Cyt C*	F:AAAGGGAGGCAAGCACAAGACTG	R:TCCAGGTACGCCATCAGTGTCTC
*Mfn1*	F:AGAAAGCACAAAGCACAGGGGATG	R:CACTGCTGACTGCGAGATACACTC
*Mfn2*	F:GCCACACCACCAACTGCTTCC	R:TCTTGACGCTCCTCTTCTCCTCTG
*Drp1*	F:TCTGAATCTGGTGGGCATGATTGC	R:CTCCGCAGTAAAGGACTCGAAGTG
*Opa1*	F:CCAACGCCATCATACTGT	R:TTCTCTGCCAGGTCTAACTT
*GAPDH*	F:ACCCAGAAGACTGTGGATGG	R:AAGCAGGGATGATGTTCTGG

## Data Availability

The data presented in this study are available on request from the corresponding author due to restrictions related to proprietary raw data.

## Supplementary Materials

The following supporting information can be downloaded at: https://www.mdpi.com/article/10.3390/vetsci13080798/s1, Table S1. List of abbreviations used in the manuscript; File S1: Original images of Western blotting.
