# Supplementary material for: PTD-FNK Alleviated Heat Stress-Induced Apoptosis of Boar Sertoli Cells via the PI3K/AKT Pathway
Source: Vet Sci. 2026 Aug 12;13(8):798. doi: 10.3390/vetsci13080798 (PMC13517425; doi:10.3390/vetsci13080798)

figure5G-actin

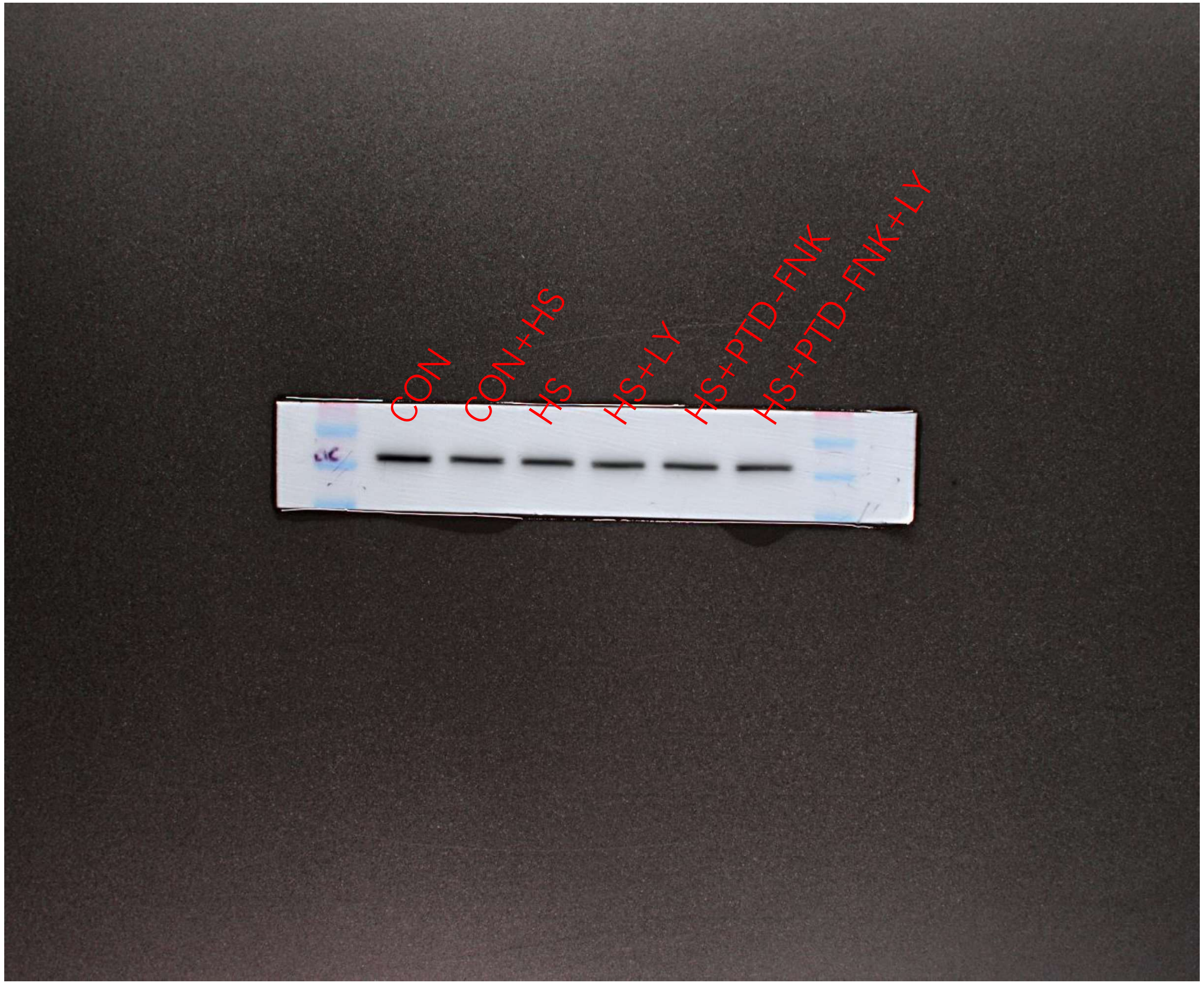

figure5G-AKT2

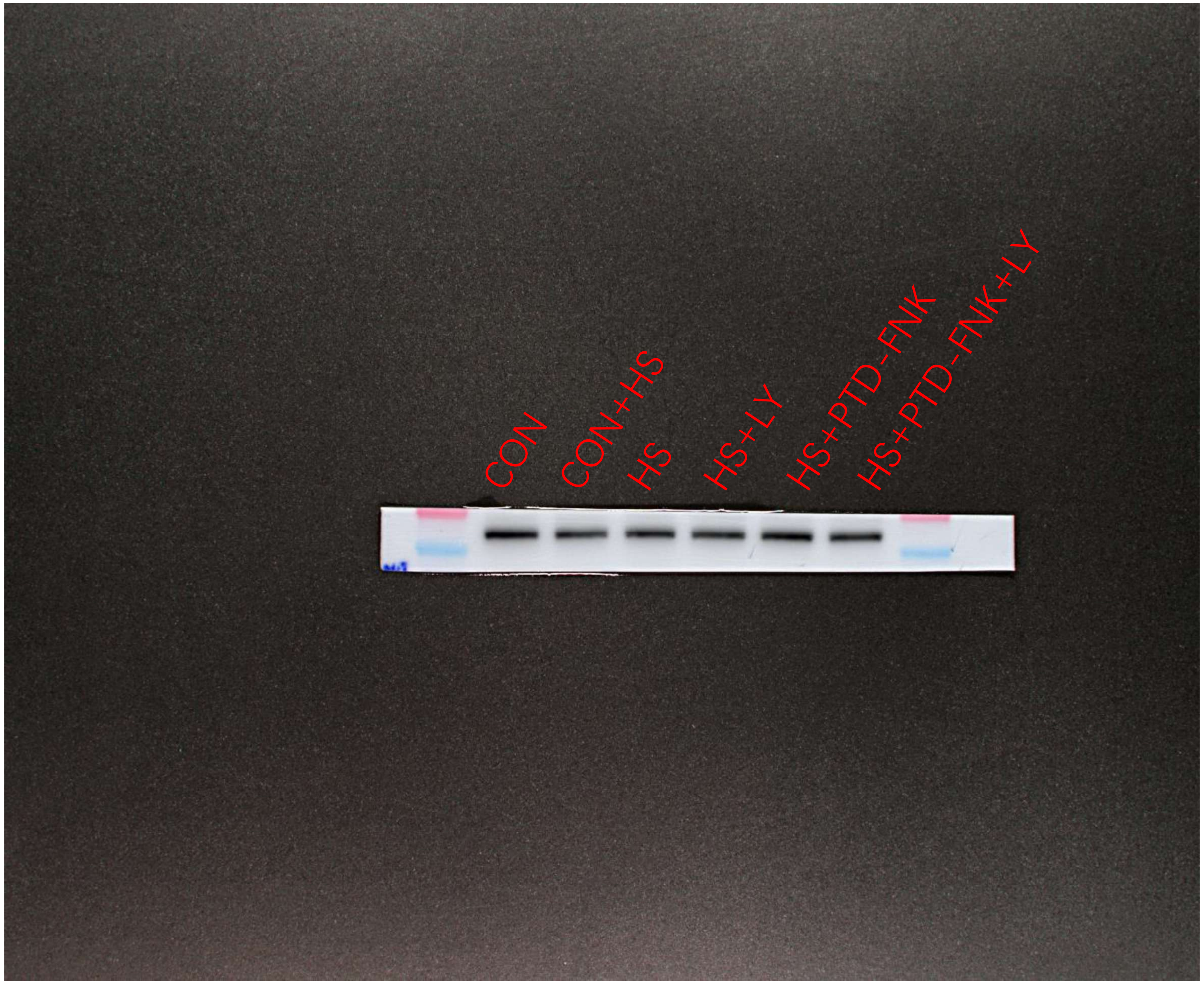

figure5G-bax

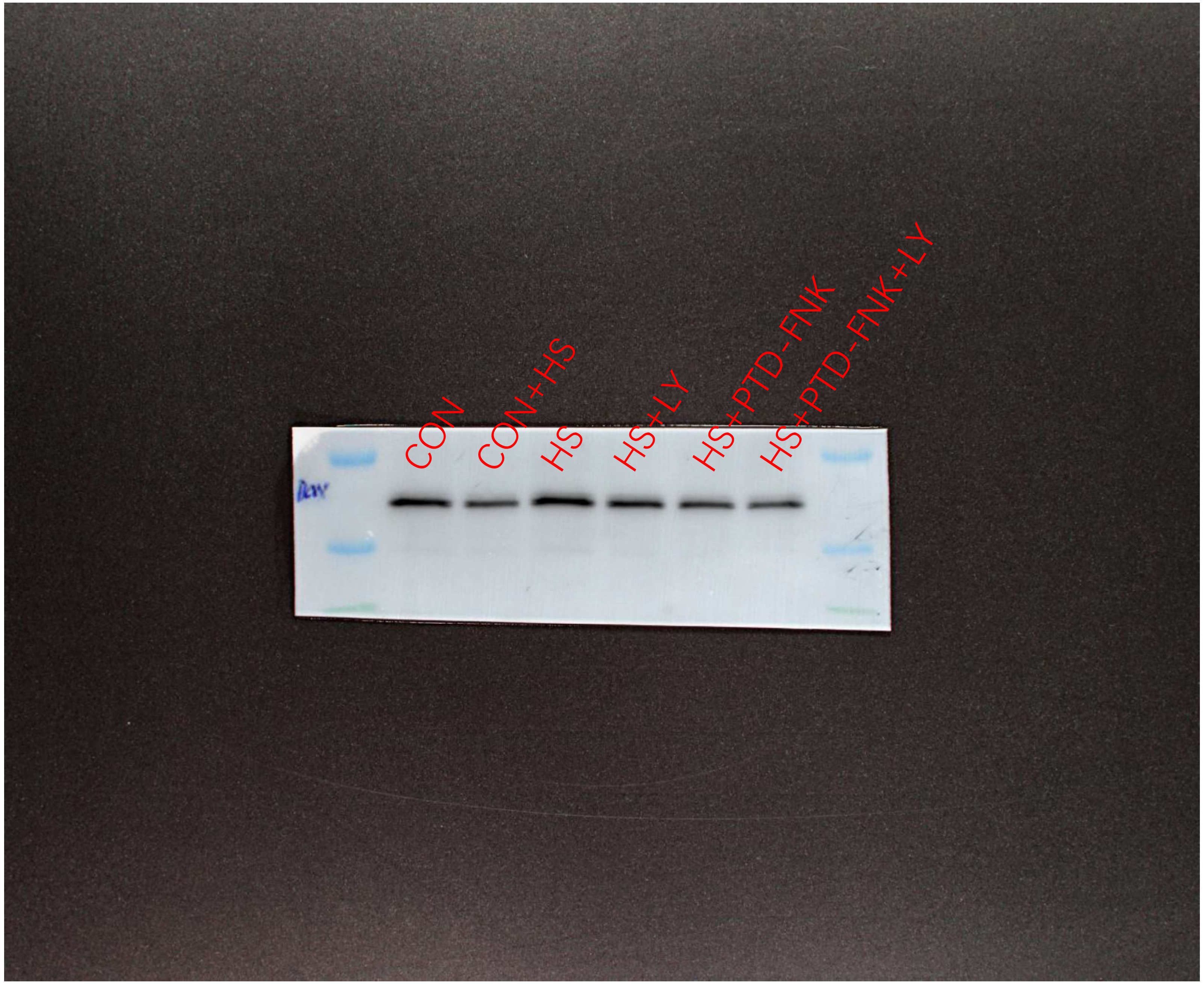

figure5G-BCL2

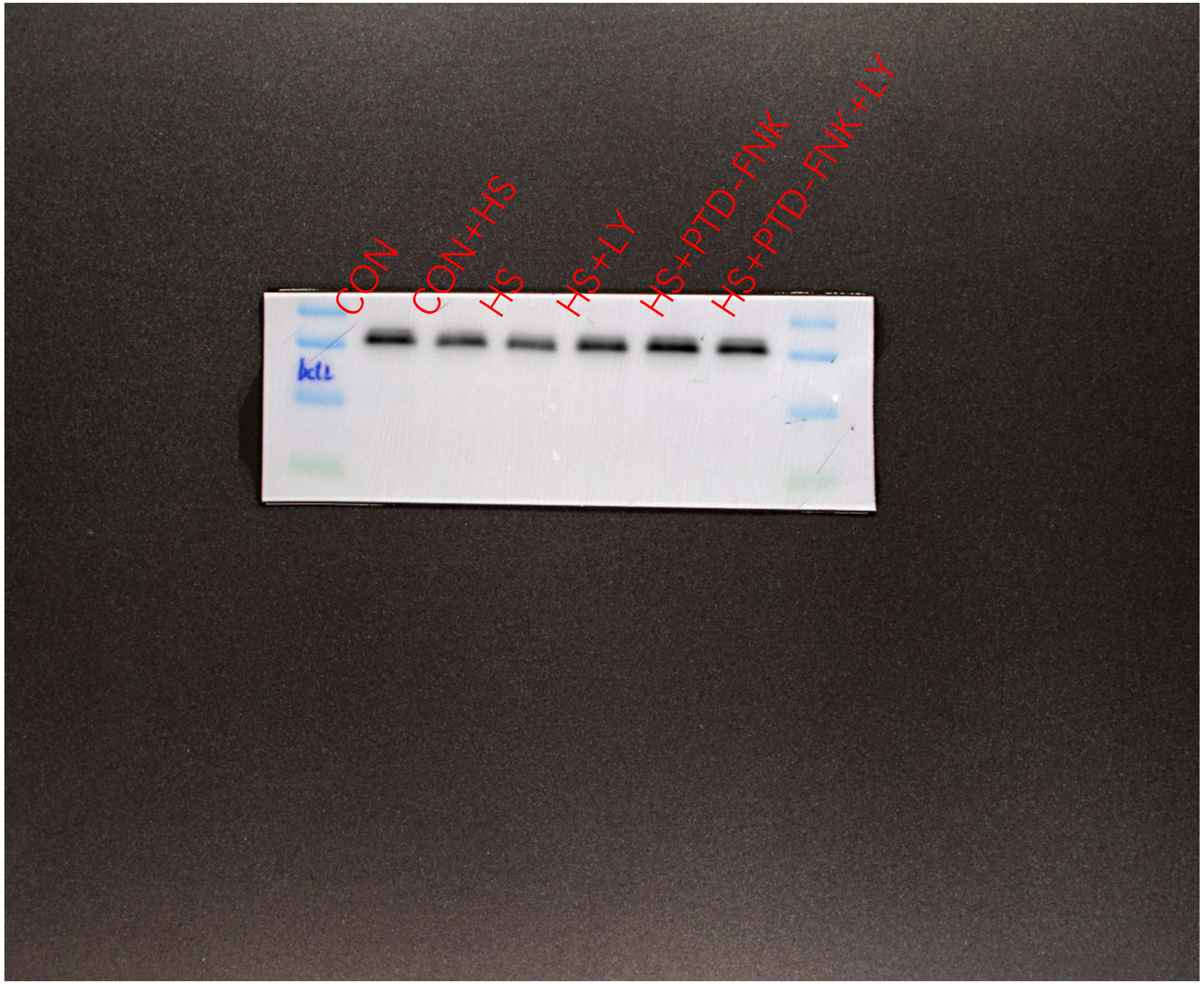

figure5G-P-AKT

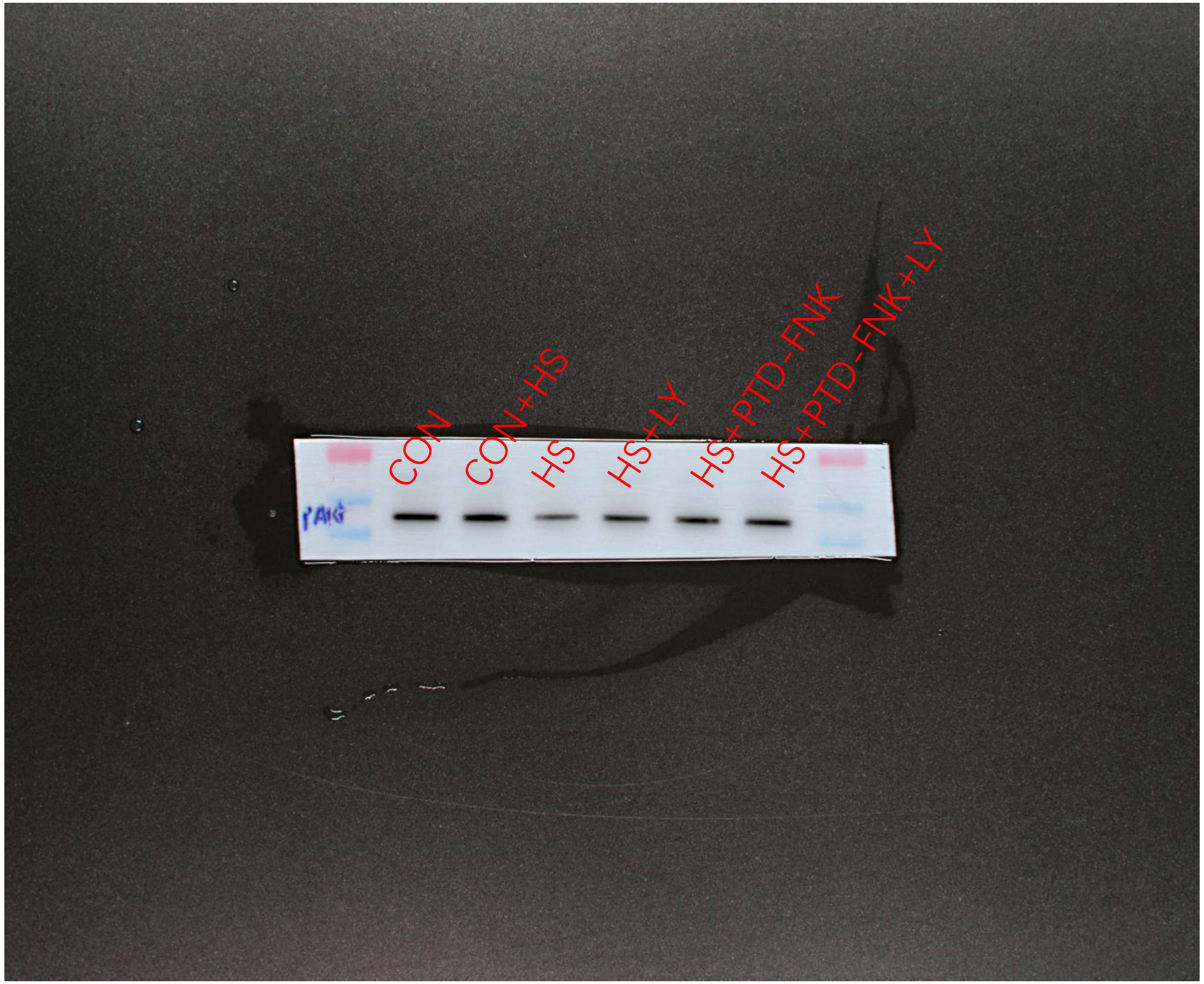

figure5G-PI3K

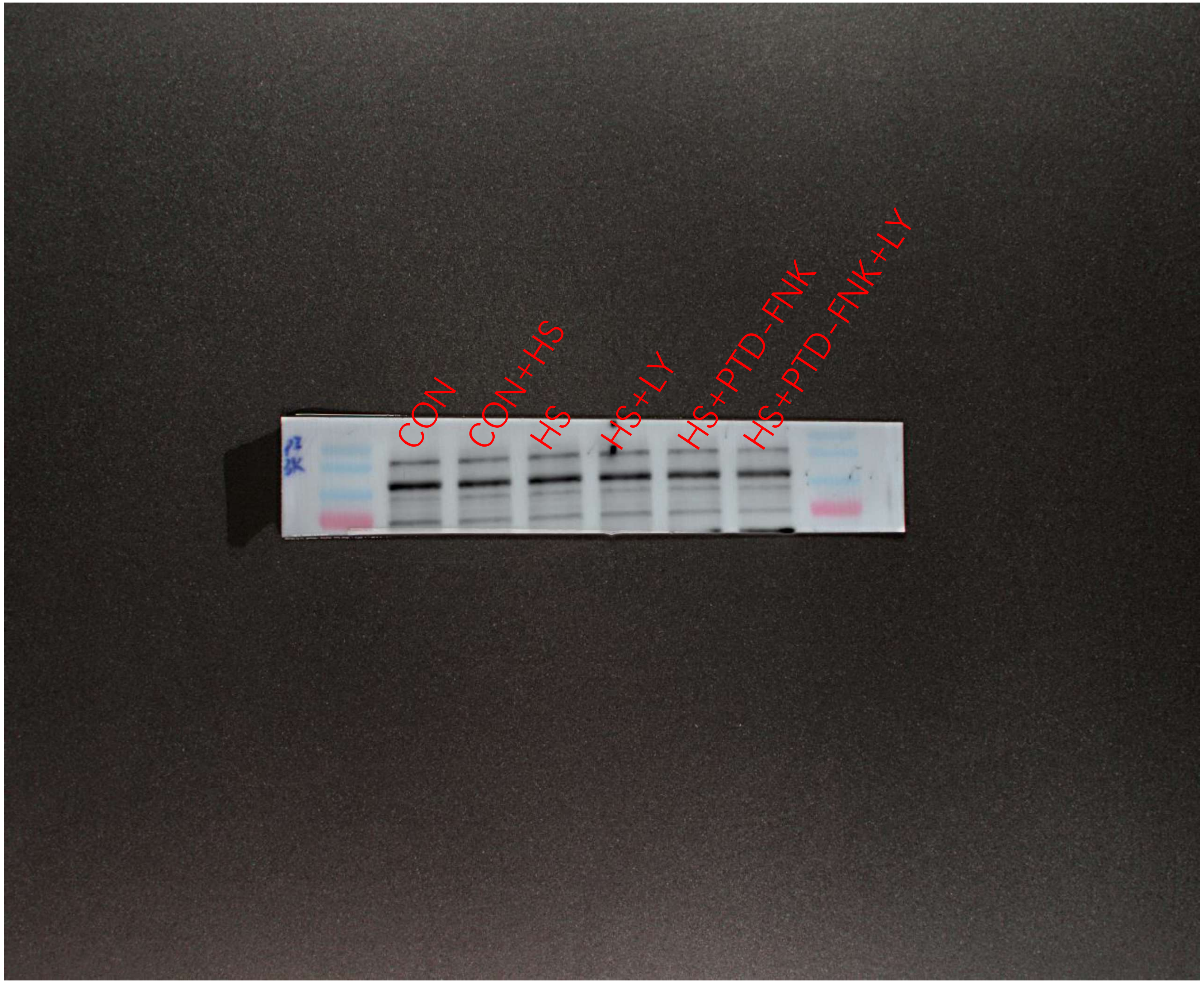

figure5G-P-PI3K

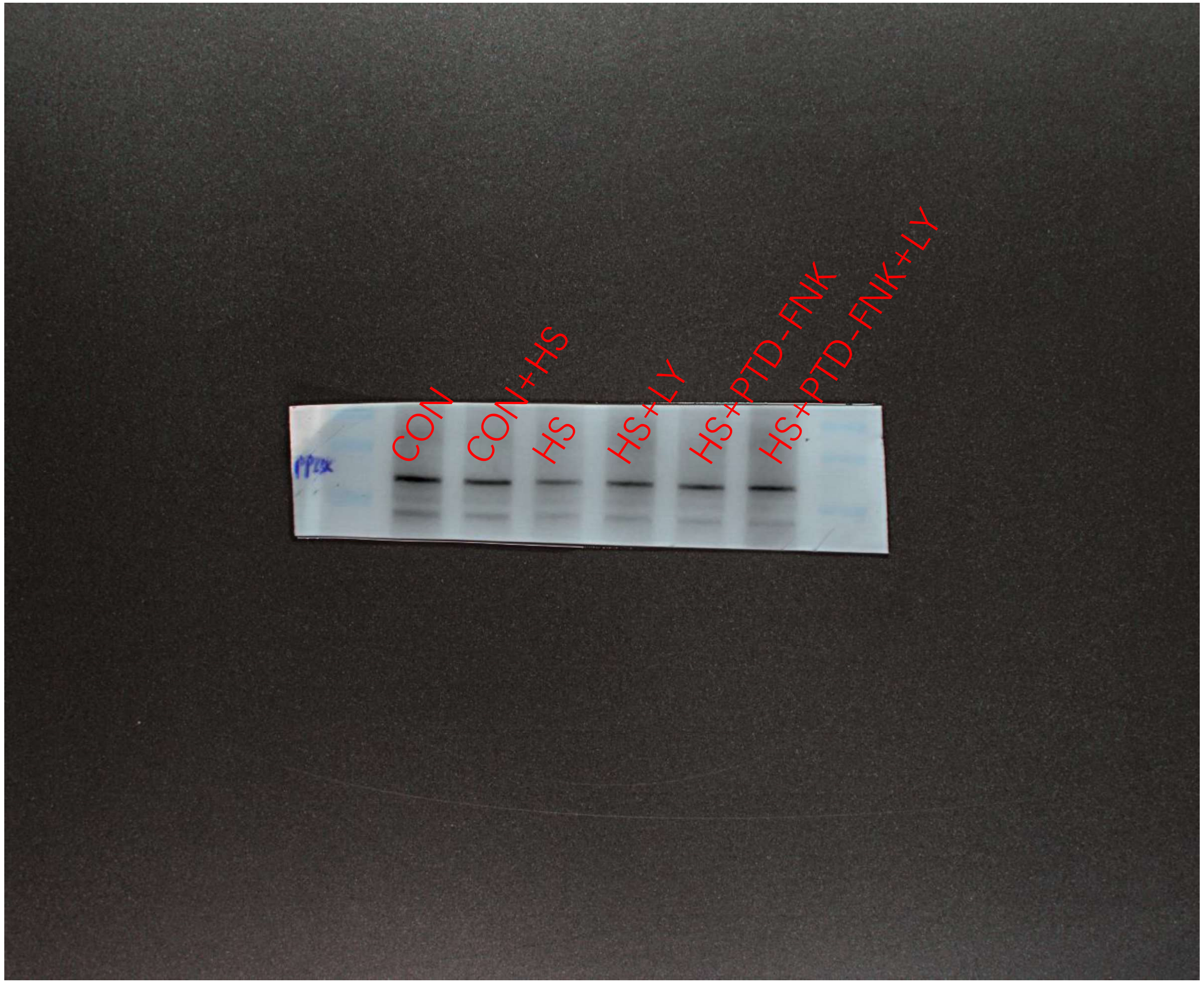

Supplement: Supplementary file 1 [file vetsci-13-00798-s001.zip › vetsci-4456558,File S1.pdf]
