# Supplementary material for: PTD-FNK Alleviated Heat Stress-Induced Apoptosis of Boar Sertoli Cells via the PI3K/AKT Pathway
Source: Vet Sci. 2026 Aug 12;13(8):798. doi: 10.3390/vetsci13080798 (PMC13517425; doi:10.3390/vetsci13080798)
Supplement: Supplementary file 1 [file vetsci-13-00798-s001.zip › vetsci-4456558,Table S1.pdf]

Table S1. List of abbreviations used in the manuscript

Abbreviation	Full Name
PTD	Protein Transduction Domain
FNK	Triple mutant of rat Bcl-x_L (Y22F/Q26N/R165K)
PTD-FNK	Protein Transduction Domain fused FNK recombinant fusion protein
HS	Heat stress
SCs	Sertoli cells
BTB	Blood-testis barrier
PI3K	Phosphatidylinositol 3-kinase
AKT	Protein kinase B
p-PI3K	Phosphorylated PI3K
p-AKT	Phosphorylated AKT
LY / LY294002	Specific PI3K inhibitor LY294002
T-AOC	Total antioxidant capacity
SOD	Superoxide dismutase
MDA	Malondialdehyde
ROS	Reactive oxygen species
MMP	Mitochondrial membrane potential
Cyt C	Cytochrome C
DEGs	Differentially expressed genes
qRT-PCR	Quantitative real-time polymerase chain reaction
RNA-seq	RNA sequencing
GO	Gene Ontology
KEGG	Kyoto Encyclopedia of Genes and Genomes
HE	Hematoxylin-Eosin
DAPI	4′,6-diamidino-2-phenylindole
FITC	Fluorescein isothiocyanate
HSP70	Heat shock protein 70
ZO-1	Zonula Occludens-1
Cx43	Connexin 43
Mfn1/2	Mitofusin 1/2
Drp1	Dynamin-related protein 1
Opa1	Optic atrophy 1
Bax	Bcl-2 Associated X Protein
Bcl-2	B-cell lymphoma-2
Caspase-3/8/9	Cysteinyl aspartate specific proteinase-3/8/9
AR	Androgen receptor
WT1	Wilms' Tumor 1
GATA4	GATA binding protein 4
CON	Control group
PBS	Phosphate Buffered Saline
DMEM	Dulbecco’s Modified Eagle Medium
FBS	Fetal Bovine Serum
PS	Penicillin-Streptomycin
ELISA	Enzyme-Linked Immunosorbent Assay
CCK-8	Cell Counting Kit-8
SDS-PAGE	Sodium Dodecyl Sulfate-Polyacrylamide Gel Electrophoresis
PVDF	Polyvinylidene Difluoride
ECL	Enhanced Chemiluminescence
HRP	Horseradish Peroxidase

ANOVA	Analysis of Variance
SRA	Sequence Read Archive
ORO	Oil Red O
